# Frequency and Severity of Ear–Nose–Throat (ENT) Symptoms during COVID-19 Infection

**DOI:** 10.3390/medicina58050623

**Published:** 2022-04-29

**Authors:** Natalia Zięba, Grażyna Lisowska, Adam Dadok, Joanna Kaczmarek, Grażyna Stryjewska-Makuch, Maciej Misiołek

**Affiliations:** 1 Department of Otorhinolaryngology and Laryngological Oncology in Zabrze, Medical University of Silesia, 41-800 Zabrze, Poland; grazyna.lisowska@onet.pl (G.L.); adamdadok@interia.pl (A.D.); msmisiolek@gmail.com (M.M.); 2 Student Society Affiliated with the Department of Otorhinolaryngology and Laryngological Oncology in Zabrze, Medical University of Silesia, 41-800 Zabrze, Poland; as029@wp.pl; 3 Department of Laryngology and Laryngological Oncology, Independent Public Research Hospital No. 7 of the Silesian Medical University in Katowice, Upper Silesian Medical Centre, 40-635 Katowice, Poland; makuch_mg@wp.pl

**Keywords:** COVID-19, ENT manifestation in COVID-19, taste disturbance, olfactory disorders, cacosmia, anosmia, dizziness in COVID-19, hearing disorders in COVID-19

## Abstract

*Background and Objectives*: Coronavirus disease 2019 (COVID-19) is a new disease entity caused by the severe acute respiratory syndrome coronavirus 2 (SARS-CoV-2). The main symptoms of infection at the onset of the pandemic include dyspnea, cough and high fever. Ear–nose–throat (ENT) symptoms are among the ones presented by patients in the course of infection. The aim of the study was to analyze the frequency of ENT symptoms and to assess their severity and duration in COVID-19 patients. *Materials and Methods*: The study included 337 patients who had been infected with SARS-CoV-2, as confirmed by a PCR test. The study participants were >18 years old; the mean age was 43.98 years ± 13.47 SD. The convalescents completed a questionnaire that contained 26 questions, including 9 detailed questions related to ENT symptoms, such as sore throat, vertigo, dizziness, hearing disorders, olfactory disorders, taste disturbance, headache, cough and dyspnea. The severity of symptoms was assessed using a Visual Analogue Scale (VAS). *Results*: The most reported ENT symptoms were olfactory disorders, which occurred in 72% of patients. The second most frequent symptom was taste disturbance (68%), VAS = 6.79 ± 3.01. Vertigo and dizziness were reported by 34% of respondents (VAS = 4.01 ± 2.01). Tinnitus was observed in 15% of patients, VAS = 3.87 ± 1.98; 14% of the subjects reported hearing impairment (VAS = 3.81 ± 2.37). *Conclusions*: Symptoms related to the sense of smell, taste and hearing are some of the most common symptoms in the course of COVID-19, which is important in the therapeutic and epidemiological management of patients. Delayed diagnosis and treatment of symptoms, especially those related to the hearing organ, may result in greater permanent damage.

## 1. Introduction

COVID-19 is caused by SARS-CoV-2, which belongs to the family Coronaviridae [1,2,3,4,5,6]. Coronaviruses are ribonucleic acid (RNA) viruses, which used to be considered mild pathogens responsible for causing moderate respiratory infections.

The discovery of a highly infectious coronavirus species causing severe acute respiratory syndrome (SARS) in 2002 in China aroused concern. This pathogen led to an excessive immune response and lung damage. The mortality rate was around 10%. In 2012, there were cases of another high-mortality zoonotic disease (35%) also caused by a coronavirus—Middle East respiratory syndrome (MERS-CoV). This type of coronavirus was characterized by relatively low infectivity [2,3].

SARS-CoV-2 is also a virus of animal origin with a lower mortality rate than SARS and MERS-CoV, amounting to about 2%, but with higher infectivity. The virus reproduction rate is estimated at around 2–3, which means that one case generates about two to three new cases [5]. The basis for the diagnosis of SARS-CoV-2 infection is the reverse transcriptase polymerase chain reaction test (RT-PCR). Biological material is most often collected from the nasopharynx [4].

As we currently know, the course of COVID-19 is varied. The patient’s age, comorbidities and the virus variant are of great importance here. Regardless of the prevailing symptoms in the subsequent waves of infection, problems with the nose, throat or ear were quite frequent. The aim of this study was to analyze whether, in addition to the olfactory and taste disorders described in the literature, other ailments, such as tinnitus, vertigo/dizziness, sore throat, headache, cough or memory impairment, occurred in the course of SARS-CoV-2 infection. We were interested in the frequency of occurrence, severity and duration of symptoms and their relationship with gender, age and comorbidities. We hypothesize that ENT symptoms in the course of COVID-19 may be relevant symptoms of the disease, on the one hand, facilitating its diagnosis, and on the other, requiring specialist treatment in order to avoid long-term impairment.

## 2. Materials and Methods

A retrospective, multicenter (Department of Otorhinolaryngology and Laryngological Oncology in Zabrze; Department of Laryngology and Laryngological Oncology in Katowice) questionnaire study was carried out. It involved 337 patients over 18 years of age who had a history of SARS-CoV-2 infection, confirmed by PCR testing. The study was conducted from December 2020 to June 2021.

The questionnaire contained 26 questions. Nine questions concerned ENT symptoms, which were: sore throat, dizziness, vertigo, hearing disorders, olfactory disorders, taste disturbance, headache, cough and dyspnea.

In the case of hearing impairment, it was determined whether the condition was transient or permanent, whether it was accompanied by tinnitus and whether the patient suffered a deterioration in speech comprehension. The questions about the olfactory disorders included sub-items describing their nature (total loss of smell, deterioration of the sense of smell and cacosmia).

Some questions were related to the occurrence and severity of gastrointestinal disorders, musculoskeletal disorders, skin lesions and memory impairment.

The respondents were also asked about the severity and duration of all infection symptoms, the need for hospitalization and the use of passive or active oxygen therapy and the presence and duration of fever.

The answers to the questions were recorded using 10-point VAS; the most severe symptoms were scored as 10 points, and no symptoms—0 points. It was assumed that the range of points 0–3 referred to mild symptoms, 4–6—moderate and 7–10—severe.

Statistical analysis: Percentages and means with standard deviations are reported in this study. The influence of demographic and medical factors on the probability of specific ENT symptoms was analyzed using logistic regression analysis. Effects significant at *p* < 0.05 are reported in the paper. Standard errors in the models were calculated by the Huber–White method. All the reported correlations are tetrachoric—appropriate for calculating the relationship between binary variables (here: presence or absence of a given symptom). The analyses were performed using Stata MP 17.0 software.

## 3. Results

The study was based on the analysis of questionnaires completed by 337 patients, including 173 females. The study participants were 18 to 86 years old. The mean age of the study group was 43.98 years ± 13.47 SD.

The most common ENT symptoms reported in the course of COVID-19 were olfactory disorders, which occurred in 72% of patients (n = 334). Complete loss of smell (44% of patients) lasted on average 15.69 (±17.84) days and occurred on average 3–4 days after the first COVID-19 symptom. A total of 23% of respondents reported that they had partial loss of smell in the course of infection, the average severity of which was estimated at 5.69 (±2.89) on the VAS scale. In 78% of cases, a complete recovery of the sense of smell was observed, and in 19%—partial.

The least common olfactory disorder reported by patients was cacosmia—it was reported by 11%, and its mean severity was 6.72 (±3.03) points.

The second most frequent ENT symptom reported by patients was taste disturbance, which occurred in 160 respondents, i.e., 68% (n = 332). The mean severity of this symptom was estimated at 6.79 (±3.01) on the VAS scale. In 65% of respondents, taste disturbance disappeared completely. The mean duration of taste disturbance was 12.88 (±13.84) days.

Headache and sore throat were also reported by some patients in the course of COVID-19. They occurred in 65% and 39% of patients, and their mean severities were 5.37 (±2.41) and 4.27 (±2.16) on the VAS scale, respectively. Runny nose occurred in 39% of respondents, and its severity was 4.35 (±2.28) on the VAS scale.

Vertigo was a significant factor that affected the patients’ quality of life. This was reported by 34% of the patients. The severity of the symptoms was rated as 4.01 (±2.01) on the VAS scale, and the mean duration of dizziness was 8 days. Vertigo ceased completely in 78% of cases, and partially in 17%.

Tinnitus was reported relatively rarely (15% of patients). It was a symptom of low severity—on average 3.87 (±1.98) on the VAS scale—but it lasted for a long time—about 15 days. In 48% of cases, tinnitus resolved completely, and in 21%—partially.

Hearing disorders were reported by 14% of subjects, estimating its severity at 3.81 (±2.37) on the VAS scale. Most patients could not determine how long exactly this symptom had persisted, whereas 45% of patients reported complete resolution of the hearing loss, and 38%—partial.

**Figure 1:**
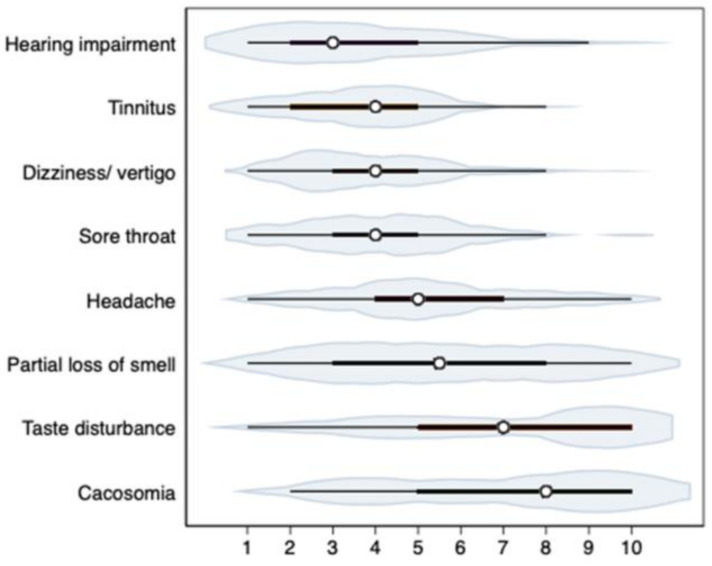
Severity of ENT symptoms assessed on the VAS scale (1–10). *Note*. The violin plots show medians (points), interquartile ranges (thick horizontal lines), ranges of 95% of observations (thin horizontal lines).

The severity of ENT symptoms assessed on the VAS scale (1–10) is shown in Figure 1.

**Figure 2:**
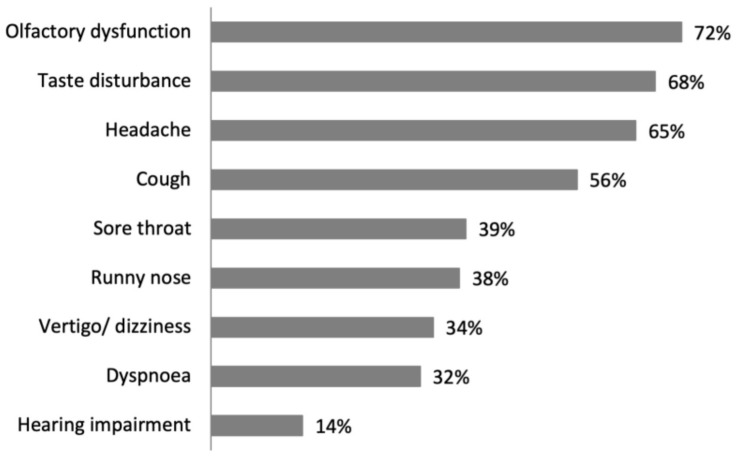
Distribution of the frequency of reported ENT symptoms.

The frequency of reported ENT symptoms is shown in Figure 2.

The analysis of demographic factors indicates the influence of gender on the occurrence of individual ENT symptoms. In the course of COVID-19, the female respondents had a higher probability of developing (or were more likely to report in the questionnaires) headaches, sore throats and vertigo/dizziness. Females reported headaches and sore throats 1.77 times more often and vertigo/dizziness 1.45 times more often than men.

Among women, ENT symptoms were slightly more frequent than in men, but the difference was not significant. Age was significantly related to the frequency of smell disorders. With age (every additional 10 years of age), the chance of developing olfactory disorders decreased by 30%.

The impact of comorbidities on the frequency of ENT symptoms in the course of COVID-19 was also analyzed. It was noteworthy that people with type 2 diabetes had a 70% lower chance of developing olfactory disorders and a 74% lower chance of developing headaches.

**Figure 3:**
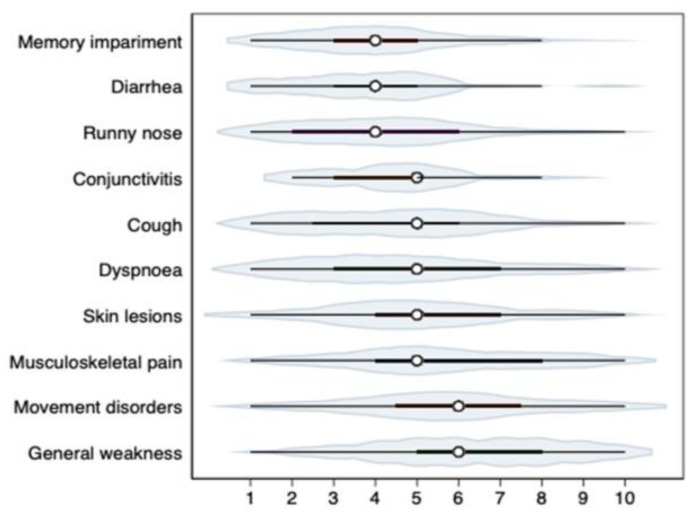
Severity of systemic symptoms assessed on the VAS scale (1–10). *Note*. The violin plots show medians (points), interquartile ranges (thick horizontal lines), ranges of 95% of observations (thin horizontal lines).

Cough and dyspnea may be ENT symptoms, although in the course of COVID-19, they are often due to lower respiratory tract involvement. Cough occurred in the course of infection in 56% of patients and had a mean severity of 4.79 on the VAS scale, whereas dyspnea occurred in 32% of respondents (VAS: 4.83). The severity of systemic symptoms assessed on the VAS scale (1–10) is shown in Figure 3.

**Figure 4:**
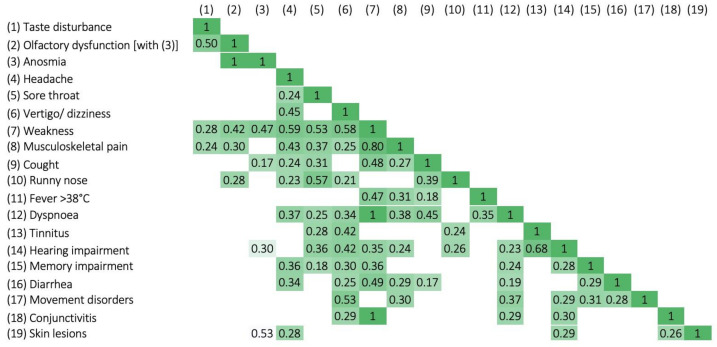
Correlation coefficient (r) of ENT and systemic symptoms in the course of COVID-19 at the significance level of *p* < 0.1.

In our study, we also analyzed the correlation coefficient (r) of ENT and systemic symptoms in the course of COVID-19 at the significance level of *p* < 0.1. The mean correlation (r = 0.68) was found in the case of hearing impairment and tinnitus, as well as between taste disturbance and olfactory disorder (r = 0.5). Low correlation (r = 0.45) occurred in the case of headache and vertigo. In the case of the other ENT symptoms, the correlation coefficient was low or there was no correlation (Figure 4).

## 4. Discussion

In our study, it was found that the most commonly reported ENT symptom occurring in patients during and after SARS-CoV-2 infection in the period from December 2020 to June 2021 was an olfactory disorder. Olfactory dysfunction occurred in 72% of respondents, and in as many as 44%, there was a complete loss of smell lasting approximately 16 days. In 19% of patients, improvement in smell was only partial. Overall, 11% of respondents reported moderate cacosmia.

The second most frequent ENT symptom reported by the patients was taste disturbance, which occurred in 68% of patients, whose severity was assessed as moderate on the VAS scale. Unfortunately, the disturbance remained partially or permanently in 35% of respondents. This is confirmed by reports from a study conducted by Korkmaz et al., based on observations of 116 hospitalized patients in whom the most common ENT symptoms were impairment or loss of taste, occurring in 41.37% of respondents, olfactory disturbances and complete loss of smell (37 and 9% of patients, respectively) and, finally, headache, reported by 37.1% of patients [7].

Chemosensory disorders (taste and smell) are frequent and early symptoms of coronavirus infection and are largely the first symptoms or the only symptoms of infection [7,8,9]. In the group of patients we analyzed, taste and olfactory disorders coexisted in 208 out of 330 patients, which is 63%, and complete loss of smell was present in the early stages of the disease. The literature often suggests that sudden loss of smell is a pathognomonic symptom of SARS-CoV-2 infection [6,8,9,10,11,12,13,14]. Smell dysfunction is more common among European patients than Asian ones [6]. European otorhinolaryngologists note that the loss of smell and taste is not usually accompanied by a runny nose or nasal obstruction. In our study, runny nose was also not correlated with the loss of smell (rho = 0.023; *p* = 0.902).

The pathogenesis of anosmia in COVID-19 is not a direct result of nasal obstruction but seems to be associated with damage to the olfactory epithelium [15,16]. Vaira et al. found that taste and smell disorders most often occur in the early stages of the disease and gradually disappear [12]. Therefore, they believe that the continuous improvement of the chemosensory function suggests that the pathogenetic mechanism causing the dysfunction of taste and smell is most likely associated with the interference of the virus with the taste and smell receptors or with local inflammation, and not with the invasion of the central nervous system and permanent damage to neurons [12]. In our study, in about 7.2% of patients, the disturbances in taste and smell were permanent (they persisted after 60 days of observation).

Our study confirms the transient nature of these disorders in most patients, 78% of whom regained the sense of smell completely, and 19% partially. Similarly, in the study by Lechien et al., 79.5% of patients regained olfactory function [17].

Petrocelli et al. [14] conducted a study in 300 COVID-19 patients. Taste and smell disorders occurred in 70% of patients. Complete loss of smell was found in 47%, and complete loss of taste in 38%. Taste disturbance was more common in women [14]. Lechien et al. [13] report that 85.6% of respondents had an olfactory disorder and that the smell dysfunction occurred before the onset of other symptoms in about 12%, after the first symptoms of the disease in about 65% of patients or simultaneously with other symptoms in about 23%. They emphasize that anosmia is a useful early marker for testing for SARS-CoV-2 or self-isolation [13].

Petrocelli et al. [14] conducted a study in 300 COVID-19 patients. Taste and smell disorders occurred in 70% of patients. Complete loss of smell was found in 47%, and complete loss of taste in 38%. Taste disturbance was more common in women [14]. Lechien et al. report that 85.6% of respondents had an olfactory disorder and that the smell dysfunction occurred before the onset of other symptoms in about 12%, after the first symptoms of the disease in about 65% of patients or simultaneously with other symptoms in about 23%. They emphasize that anosmia is a useful early marker for testing for SARS-CoV-2 or self-isolation [13].

Vaira et al. [12] believe that the loss of taste in COVID-19 is not directly related to an olfactory disorder. They list two mechanisms by which the loss of taste is most likely to occur. According to the first one, the virus damages cells by using angiotensin-converting enzyme-2 (ACE-2) receptors located on the taste buds. By binding the receptors, SARS-CoV-2 may inactivate them, making it difficult to change the chemical taste stimuli into an action potential, thus preventing taste perception [12]. The other mechanism involves the binding of SARS-CoV-2 to sialic acid receptors. Sialic acid is the basic component of mucin in saliva and protects glycoproteins, which transport flavor particles into the flavor pores prior to premature enzymatic degradation. Reducing the amount of sialic acid in saliva is associated with an increase in taste threshold. SARS-CoV-2 may occupy sialic acid binding sites on the taste buds, accelerating the degradation of taste particles [8]. SARS-CoV-2 is detected in saliva in the early stage of the disease and in saliva collected from the orifice of the salivary gland ducts, as well as in the later stage [2].

Headache, sore throat and runny nose were other symptoms reported by some COVID-19 patients. Vertigo is also one of the symptoms relatively frequently reported. In our study, it occurred in 34% of patients, was of moderate intensity and lasted about 8 days. In 78% of cases, the dizziness resolved completely, and in 17%—partially. Early research from China found that vertigo was the most common neurological manifestation of the disease [18]. SARS-CoV-2 is known to have neuroinvasive potential [18,19,20]. Other possible systemic causes of vertigo in the course of this infection include hypoxia, thromboembolic complications or immunological background [18,20]. The presence of dizziness may also be directly related to acute labyrinthitis, vestibular neuritis or acute otitis media occurring in the course of COVID-19 infection [18,21].

In the study by Korkmaz et al., vertigo was similarly as frequent as in our study (31.8% vs. 34%, respectively) [7]. The questionnaire used in their study asked about the occurrence of vertigo in the course of COVID-19 infection. These symptoms were indicated by 34% of respondents. Objective tests were not performed, and it was difficult for patients to clarify the nature of vertigo; therefore, there is no information detailing whether the patients experienced peripheral or central vertigo. The authors found a weak correlation between the occurrence of vertigo and headaches. The complaints reported less frequently by our patients (15% of respondents) included tinnitus; their intensity was on average 3.87 (±1.98) on the VAS scale, but they lasted for a long time, i.e., for about 15 days. Unfortunately, the tinnitus only completely resolved in 48% of patients, and in 21%—partially.

COVID-19 infection may have a detrimental effect on the cochlear outer hair cells, despite the asymptomatic or mildly symptomatic course of the disease [22,23]. In Mustafa’s study, damage to the outer hair cells was confirmed by the reduced amplitude of snap-induced otoacoustic emissions in the test group compared to the control group, and by tonal audiometry [22]. Hearing loss, which occurred within 3–4 weeks after the onset of COVID-19, is considered to be associated with this infection, but it requires careful medical interview analysis and additional tests [24].

In our study, 14% of respondents reported hearing deterioration; unfortunately, only 45% reported a complete improvement in hearing after the infection was over. Such a large percentage of patients with permanent hearing or balance disorders should be a strong argument for conducting early ENT/audiological diagnostics in COVID-19 patients. The obtained mean (r = 0.68) correlations in the case of hearing deterioration and tinnitus as well as between taste and olfactory disorders (r = 0.5) seem interesting, which may suggest significant viral neurotropism.

In a study involving 1420 patients, Lechien et al. noted differences in the incidence of COVID-19 symptoms depending on gender and age. Young people more often manifest nasal and sinus symptoms, nasal obstruction, runny nose, headache and sore throat. Loss of smell is also more common in this group. The elderly complain primarily of fatigue, loss of appetite, fever and diarrhea. Women reported loss of smell, nasal obstruction, headache, sore throat and fatigue more often than men who reported cough and fever more frequently [10]. Similar conclusions can be drawn from our study, which shows that age is significantly correlated with the occurrence of olfactory disorders, which are more common in younger people. Korkmaz et al. also noted that tinnitus, peripheral dizziness, headaches, olfactory and taste disturbances, sore throat and voice disorders were statistically significantly more common in patients under 60 years of age. On the other hand, dyspnea and cough were more common in patients over 60 years of age [7].

Due to the frequency of ENT symptoms in the course of COVID-19, it should be taken into account that a patient visiting otorhinolaryngology clinics with symptoms such as taste disturbances, olfactory disturbances, runny nose, dyspnea, headache, nasal obstruction, sore throat or even tinnitus and hearing impairment may potentially be infected with SARS-CoV-2 [24], which requires appropriate therapeutic management.

Professor Hopkins and Kumar of the Rhinology Society recommend that oral corticosteroids should not be included in the treatment of emerging anosmia, as they may exacerbate COVID-19 infection. Similarly, nasal steroids are not recommended in the case of sudden loss of smell [4]. Patients suffering from infection should be treated symptomatically. However, the symptoms that persist after recovering from SARS-CoV-2 remain a problem. Due to the usually transitory nature of taste and olfactory disorders, Vaira et al. point out that the treatment of these symptoms should be started 20 days after the disease onset. The authors note that oral steroids improve smell function after viral infection, but in the case of COVID-19, which carries a risk of acute respiratory failure, there are concerns about their use. In the treatment of olfactory disorders, alpha-lipoic acid, supplementation with Omega-3 and nasal preparations with vitamin A used together with olfactory training may prove effective [12].

However, there are no clear guidelines on how to deal with patients with persistent taste and olfactory disorders, vertigo, hearing loss or tinnitus. The treatment of the ENT consequences of COVID-19 will remain an important issue in the coming years.

The limitations of our study were not carrying out objective smell and taste tests, hearing tests and balance tests on the patients. All collected data were based on subjective symptoms reported by patients. Conducting objective tests for research at that time was not recommended due to the high risk of transmission of COVID-19. A study based on objective tests of ENT symptoms associated with COVID-19 would be very valuable, and it needs further investigation when the epidemiological risk is lower.

## 5. Conclusions

1. ENT symptoms are some of the most common COVID-19 symptoms. Age was significantly related to the frequency of smell disorders. With age (every additional 10 years of age), the chance of developing olfactory disorders decreased by 30%.
2. Vertigo/dizziness were less frequent but were significant factors affecting the patients’ quality of life.
3. Apart from olfactory and taste disorders, careful attention should be paid to patients with sore throats, sudden dizziness of unknown origin and tinnitus.
4. Correct diagnosis will help to avoid therapeutic errors and symptom consolidation. Maintaining constant epidemiological vigilance is of great importance here.

## References

[1] Islamoglu Y., Gemcioglu E., Ates I. (2020). Objective evaluation of the nasal mucosal secretion in COVID-19 patients with anosmia. Ir. J. Med Sci..

[2] Freni F., Meduri A., Gazia F., Nicastro V., Galletti C., Aragona P., Galletti C., Galletti B., Galletti F. (2020). Symptomatology in head and neck district in coronavirus disease (COVID-19): A possible neuroinvasive action of SARS-CoV-2. Am. J. Otolaryngol..

[3] El-Anwar M.W., Elzayat S., Fouad Y.A. (2020). ENT manifestation in COVID-19 patients. Auris Nasus Larynx.

[4] Krajewska J., Krajewski W., Zub K., Zatoński T. (2020). COVID-19 in otolaryngologist practice: A review of current knowledge. Eur. Arch. Oto-Rhino-Laryngol..

[5] Grag K., Shubhanshu K. (2020). Effect of Covid-19 in Otorhinolaryngology Practice: A Review. Indian J. Otolaryngol. Head Neck Surg..

[6] Lechien J.R., Chiesa-Estomba C.M., De Siati D.R., Horoi M., Le Bon S.D., Rodriguez A., Dequanter D., Blecic S., El Afia F., Distinguin L. (2020). Olfactory and gustatory dysfunctions as a clinical presentation of mild-to-moderate forms of the coronavirus disease (COVID-19): A multicenter European study. Eur. Arch. Oto-Rhino-Laryngol..

[7] Korkmaz M., Eğilmez O.K., Özçelik M.A., Güven M. (2020). Otolaryngological manifestations of hospitalised patients with confirmed COVID-19 infection. Eur. Arch. Oto-Rhino-Laryngol..

[8] Vaira L.A., Lechien J.R., Salzano G., Salzano F.A., Maglitto F., Saussez S., De Riu G. (2020). Gustatory Dysfunction: A Highly Specific and Smell-Independent Symptom of COVID-19. Indian J. Otolaryngol. Head Neck Surg..

[9] Da Costa K.V., Carnaúba A.T.L., Rocha K.W., de Andrade K.C.L., Ferreira S.M., Menezes P.D.L. (2020). Olfactory and taste disorders in COVID-19: A systematic review. Braz. J. Otorhinolaryngol..

[10] Lechien J.R., Chiesa-Estomba C.M., Place S., Van Laethem Y., Cabaraux P., Mat Q., Huet K., Plzak J., Horoi M., Hans S. (2020). Clinical and epidemiological characteristics of 1420 European patients with mild-to-moderate coronavirus disease 2019. J. Intern. Med..

[11] Saussez S., Lechien J.R., Hopkins C. (2020). Anosmia: An evolution of our understanding of its importance in COVID-19 and what questions remain to be answered. Eur. Arch. Oto-Rhino-Laryngol..

[12] Vaira L.A., Hopkins C., Petrocelli M., Lechien J.R., Chiesa-Estomba C.M., Salzano G., Cucurullo M., Salzano F.A., Saussez S., Boscolo-Rizzo P. (2020). Smell and taste recovery in coronavirus disease 2019 patients: A 60-day objective and prospective study. J. Laryngol. Otol..

[13] Lechien J.R., Hopkins C., Saussez S. (2020). Sniffing out the evidence; It’s now time for public health bodies recognize the link between COVID-19 and smell and taste disturbance. Rhinology.

[14] Petrocelli M., Ruggiero F., Baietti A.M., Pandolfi P., Salzano G., Salzano F.A., Lechien J.R., Saussez S., De Riu G., Vaira L.A. (2020). Remote psychophysical evaluation of olfactory and gustatory functions in early-stage coronavirus disease 2019 patients: The Bologna experience of 300 cases. J. Laryngol. Otol..

[15] Zhang Q., Shan K.S., Abdollahi S., Nace T. (2020). Anosmia and Ageusia as the Only Indicators of Coronavirus Disease 2019 (COVID-19). Cureus.

[16] Lechien J.R., Barillari M.R., Jouffe L., Saussez S. (2020). Anosmia Is a Key Symptom of COVID-19 Infection and Should Be Used as a Diagnostic Tool. Ear Nose Throat J..

[17] Lechien J.R., Journe F., Hans S., Chiesa-Estomba C.M., Mustin V., Beckers E., Vaira L.A., De Riu G., Hopkins C., Saussez S. (2020). Severity of Anosmia as an Early Symptom of COVID-19 Infection May Predict Lasting Loss of Smell. Front. Med..

[18] Saniasiaya J., Kulasegarah J. (2020). Dizziness and COVID-19. Ear Nose Throat J..

[19] Karimi-Galougahi M., Naeini A.S., Raad N., Mikaniki N., Ghorbani J. (2020). Vertigo and hearing loss during the COVID-19 pandemic–is there an association?. Acta Otorhinolaryngol. Ital..

[20] Saniasiaya J. (2020). Hearing Loss in SARS-CoV-2: What Do We Know?. Ear Nose Throat J..

[21] Maharaj S., Bello Alvarez M., Mungul S., Hari K. (2020). Otologic dysfunction in patients with COVID-19: A systematic review. Laryngoscope Investig. Otolaryngol..

[22] Mustafa M. (2020). Audiological profile of asymptomatic Covid-19 PCR-positive cases. Am. J. Otolaryngol..

[23] Chirakkal P., Al Hail A.N., Zada N., Vijayakumar D.S. (2020). COVID-19 and Tinnitus. Ear Nose Throat J..

[24] Satar B. (2020). Criteria for establishing an association between Covid-19 and hearing loss. Am. J. Otolaryngol..

